# Elasnin Effectively Eradicates Daptomycin-Resistant Methicillin-Resistant Staphylococcus aureus Biofilms

**DOI:** 10.1128/spectrum.02320-21

**Published:** 2022-02-23

**Authors:** Jordy Evan Sulaiman, Lexin Long, Pei-Yuan Qian, Henry Lam

**Affiliations:** a Department of Chemical and Biological Engineering, The Hong Kong University of Science & Technology, Kowloon, Hong Kong, People’s Republic of China; b Department of Ocean Science and Hong Kong Branch of Southern Marine Science and Engineering Guangdong Laboratory, Guangzhou, The Hong Kong University of Science & Technology, Kowloon, Hong Kong, People’s Republic of China; c Southern Marine Science and Engineering Guangdong Laboratory, Guangzhou, Guangdong, People’s Republic of China; Weizmann Institute of Science

**Keywords:** MRSA, antibiotics, elasnin, resistance, proteomics, biofilms

## Abstract

Elasnin is a recently reported antibiofilm agent that is effective against Gram-positive bacteria including methicillin-resistant Staphylococcus aureus (MRSA). Remarkably, we observed that elasnin has a superior activity in eradicating daptomycin-resistant MRSA strain biofilm, with a lower minimum biofilm eradication concentration (MBEC) value of 0.625 μg/mL, compared to 2.5 μg/mL for the wild type. Confocal microscopy further confirmed the higher biofilm eradication on the daptomycin-resistant strain, displaying ∼53% decrease in cell density upon elasnin treatment, while the wild-type strain was only decreased by ∼15%. Quantitative proteomics revealed that the daptomycin-resistant strain has a lower expression of the membrane, cell wall, and extracellular proteins, and also proteins involved in the arginine biosynthesis, pathogenesis, and cell adhesion compared to the wild type, which may result in weaker biofilm development. This study highlights the potential clinical application of elasnin through its superior biofilm eradication activity against a daptomycin-resistant MRSA strain, and revealed the associated processes governing this superior activity through proteomics analysis.

**IMPORTANCE** Due to the increased use of daptomycin for the treatment of MRSA infections, the emergence of daptomycin-resistant strains has become prevalent in recent years. In this study, we discovered that elasnin, a newly reported antibiofilm compound, has a superior activity in eradicating daptomycin-resistant MRSA strain biofilms compared to the wild type. Follow-up analysis revealed the reason behind this superior activity, which is the lower expression of key proteins that play a role in pathogenesis and cell adhesion in the daptomycin-resistant strain, leading to weaker biofilm development. This showcases the potential use of elasnin in clinical settings where daptomycin-resistant strains and biofilm formation are prevalent. Altogether, our study provides new insights into the mechanism of elasnin in MRSA biofilm cells and identified its superior biofilm eradicating activity in the daptomycin-resistant strain.

## INTRODUCTION

Elasnin was first introduced as a human granulocyte elastase inhibitor that has minimal to none toxic side effects (1, 2). Several studies have suggested that elasnin could be used to treat chronic obstructive lung disease caused by leukocyte elastase that damages the lung tissue (3, 4). Recently, the antibiofilm activity of elasnin was explored, and it was reported that elasnin inhibited biofilm formation of multiple species in the marine environment, including some Gram-positive bacteria such as Staphylococcus aureus (5, 6). Further investigation showed that elasnin did not only inhibit biofilm formation, but also effectively eradicated the biofilms of methicillin-resistant Staphylococcus aureus (MRSA) through the repression of the virulence regulon and interference of the cell division process (i.e., by suppressing the production of newly synthesized extracellular polymeric substance [EPS], cell wall and membrane components, and peptidoglycan hydrolases that are needed to finish cell wall formation) (7). Since elasnin’s main mode of action is by disrupting the biofilm matrix and interfering with the cell division process, it has limited ability to kill biofilm or stationary phase cells (7). However, the resulting elasnin-treated biofilm cells were shown to exhibit cell wall defects and became more sensitive to β-lactams such as penicillin G. Follow-up analysis showed that although elasnin could barely kill non-growing stationary phase or biofilm cells upon prolonged treatment, it exhibited antibacterial activity against growing S. aureus planktonic cells, albeit with slow killing kinetics (8).

The last-resort antibiotics against MRSA bacteremia infections are vancomycin (VAN) and daptomycin (DAP) (9, 10). However, due to the extensive use of these two antibiotics, clinical isolates that are resistant to VAN and DAP were frequently reported and quickly became prevalent in clinics (11–15). In this study, we explored the effectiveness of elasnin against a daptomycin-resistant MRSA (DAP^R^) strain that was isolated from our previous work, bearing a single point mutation in the *mprF* gene that matched those observed in clinical isolates (16). This is particularly important since the DAP^R^ MRSA strain possessed membrane and other cell wall-related modifications, which were known to be affected by elasnin according to a previous study (7). Moreover, in our previous study, we have also shown that the DAP^R^ strain has defects in biofilm forming abilities compared to the wild-type (WT) MRSA, which might impact their survival toward elasnin treatment (16). Interestingly, here we discovered that elasnin has a superior biofilm eradicating activity against the DAP^R^ strain compared to the susceptible WT strain. This highlighted the potential clinical application of elasnin, since MRSA strains often formed biofilms in medical devices (17), and DAP^R^ biofilm was known to be harder to eradicate compared to the WT biofilm with either DAP or VAN (16). Using proteomics, we compared the proteome profile of DAP^R^ and the WT biofilm cells under elasnin treatment to reveal the reason behind elasnin’s superior biofilm eradication activity against the DAP^R^ strain.

## RESULTS AND DISCUSSION

### Elasnin has a superior biofilm inhibition and eradication activity against DAP^R^ MRSA compared to the WT.

It was reported that elasnin interfered with cell division and cell wall biosynthesis in both planktonic culture and biofilms (7, 8). Therefore, we were interested in seeing how elasnin affects a daptomycin-resistant MRSA (DAP^R^) strain, which has membrane and cell-wall modifications due to the mutation it possessed (T345A gain-of-function in the *mprF* gene), and also lower biofilm formation compared to the WT strain (16). Our recent transcriptomic study has also shown that elasnin treatment led to a 10-fold downregulation in the expression of *mprF* gene on MRSA planktonic cells, the gene that was mutated in the DAP^R^ strain (8).

**FIG 1:**
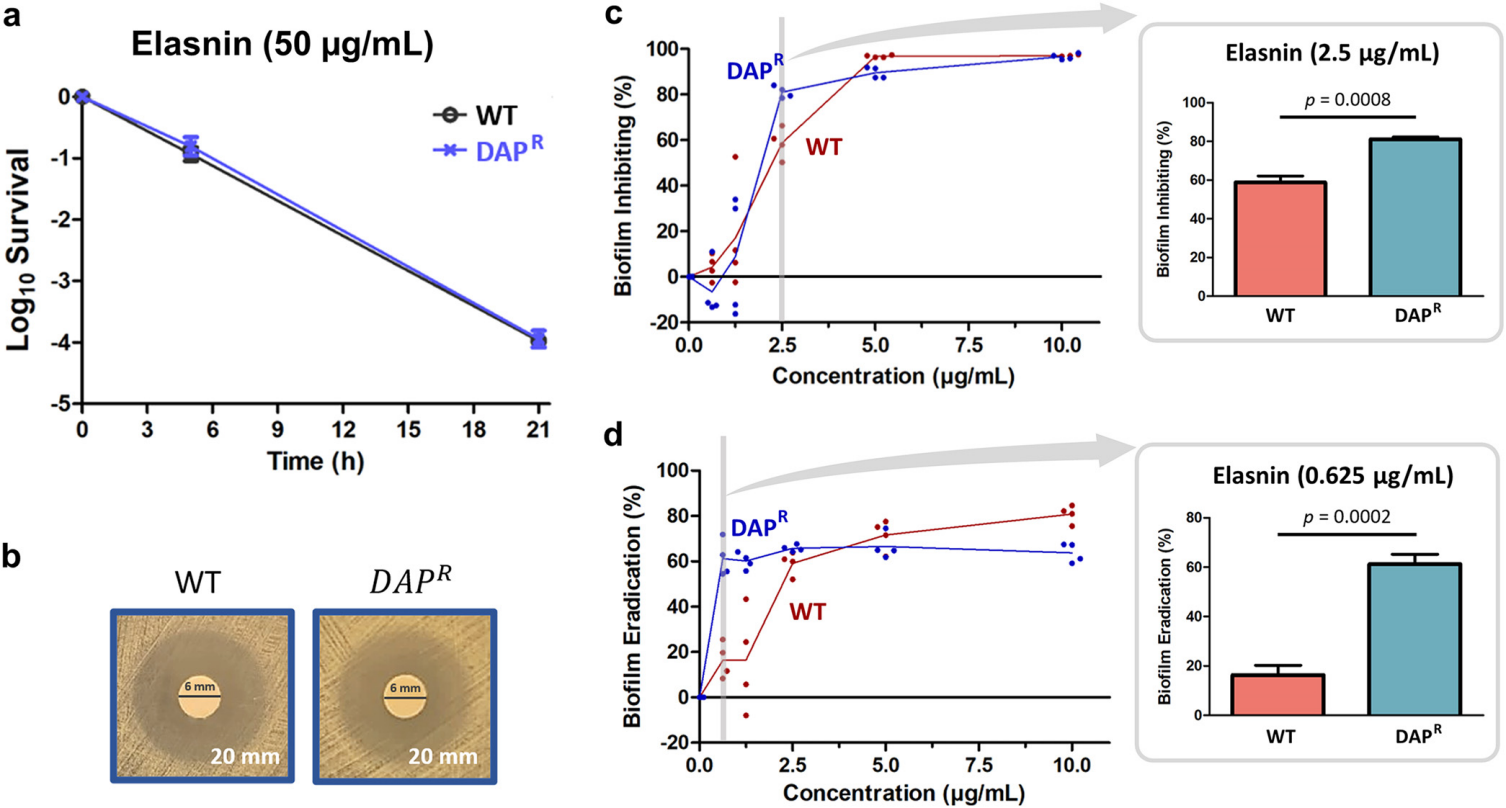
Susceptibility of WT and DAP^R^ strain planktonic and biofilm cells toward elasnin. (a) Time-kill curve of exponential-phase WT and DAP^R^ MRSA planktonic cells under elasnin treatment (50 μg/mL) (mean ± SEM, *n* = 3). (b) MIC test toward elasnin carried out using disc diffusion antibiotic sensitivity testing (disc content 40 μg). The text on the lower right corner marks the diameter of the zone of inhibitions. (c–d) Biofilm assay of the WT and DAP^R^ strain toward elasnin treatment. Antimicrobial activities of elasnin against daptomycin susceptible WT MRSA and daptomycin resistant MRSA (DAP^R^), represented by the minimum concentration needed to inhibit 90% biofilm formation (minimum biofilm inhibitory concentration, MBIC) (c) and minimum concentration needed to eradicate 50% mature biofilm (minimum biofilm eradication concentration, MBEC) (d) (mean ± SEM, *n* = 4). The figure on the right shows the biofilm inhibition % (c) and biofilm eradication % (d) of WT and DAP^R^ MRSA under 2.5 μg/mL and 0.625 μg/mL of elasnin treatment, respectively. *P* values were estimated using two-tailed Student’s *t*-test.

First, we investigated the antibacterial activity of elasnin toward planktonic culture of WT and DAP^R^ MRSA strains. Time-kill assay showed that after prolonged elasnin treatment, the survival level for WT and DAP^R^ MRSA were similar (∼0.01% survival after 21 h) (Fig. 1a), and there was no difference in the MIC toward elasnin between the WT and DAP^R^ MRSA, as measured by broth microdilution (4 μg/mL) and disc diffusion assay (Fig. 1b). These results suggested that despite the membrane and cell-wall modifications in the DAP^R^ strain that conferred its resistance, elasnin could still eliminate most of the growing DAP^R^ cells to the same extent as in the WT strain when applied at a high dose (>10× MIC). Next, we looked at the differences between elasnin treatment in the WT biofilm cells and the DAP^R^ biofilm cells. Interestingly, biofilm assay revealed that although the measured minimum biofilm inhibitory concentration (MBIC) of elasnin toward both WT and DAP^R^ MRSA were the same (5 μg/mL), the minimum biofilm eradication concentration (MBEC) of elasnin toward DAP^R^ MRSA was much lower compared to the WT (0.625 μg/mL and 2.5 μg/mL for DAP^R^ and WT, respectively) (Fig. 1c and d). These relatively low MBEC values (compared to other antibiotics such as daptomycin and vancomycin, with MBEC of 20 μg/mL [5, 16]) showcased the excellent performance of elasnin in disrupting mature biofilms not only in the WT strain but also in the DAP^R^ strain. Moreover, the activity of elasnin was observed to be higher in the DAP^R^ strain compared to the WT. Under 2.5 μg/mL of elasnin treatment, the biofilm inhibition percentage for the DAP^R^ strain was 81%, compared to 59% for the WT (Fig. 1c). Similarly, under 0.625 μg/mL of elasnin treatment, the biofilm eradication percentage for the DAP^R^ strain was 61%, compared to 16% for the WT (Fig. 1d). Since the superior activity of elasnin was more evident in biofilm eradication than in inhibition, we focused on investigating the former phenomenon. Besides, it was also reported that the DAP^R^ strain biofilm was harder to eradicate using two of the most commonly used antibiotics in clinics against MRSA, DAP, and VAN, compared to the DAP-susceptible WT (16). Therefore, elasnin’s superior biofilm eradication activity against the DAP^R^ strain would have important clinical implications.

### Confocal microscopy of the DAP^R^ MRSA strain biofilm eradication upon elasnin treatment.

**FIG 2:**
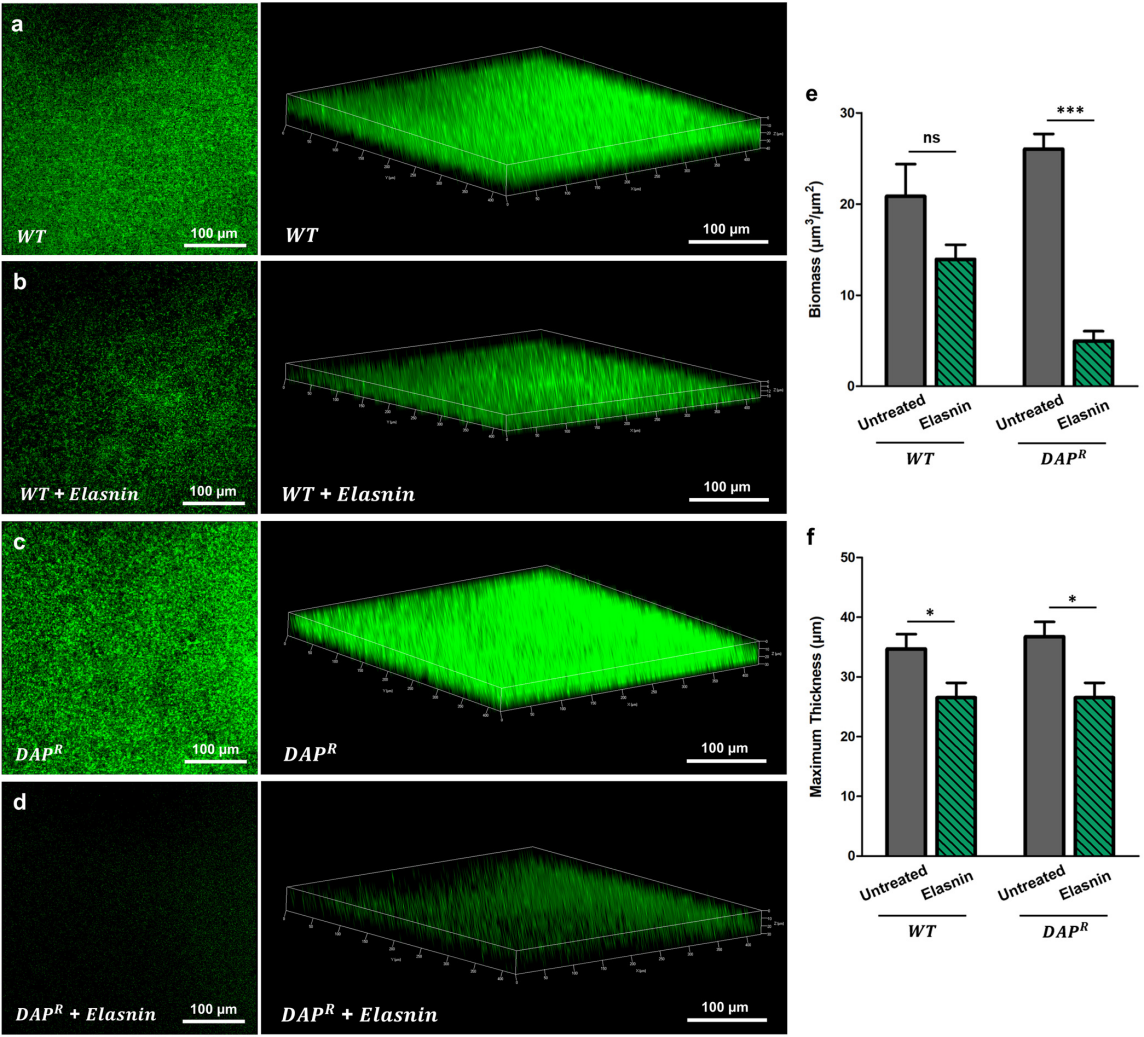
Comparison of elasnin treatment on WT and DAP^R^ MRSA biofilms using confocal microscopy. (a–d) Image of the preformed WT MRSA biofilms (24 h of incubation) after another incubation for 18 h without antibiotic treatment (a) and the preformed WT MRSA biofilms (24 h of incubation) after another incubation for 18 h with elasnin treatment at a concentration of 0.625 μg/mL (b). Image of the preformed DAP^R^ MRSA biofilms (24 h of incubation) after another incubation for 18 h without antibiotic treatment (c) and the preformed DAP^R^ MRSA biofilms (24 h of incubation) after another incubation for 18 h with elasnin treatment at a concentration of 0.625 μg/mL (d). For a–d, the left and right images are two- and three-dimensional confocal images of biofilm cells stained with FilmTracer FM 1-43 green biofilm cell stain. (e-f) Quantitative analysis of the confocal images. Biomass (e) and maximum thickness (f) of WT and DAP^R^ MRSA without and with elasnin treatment were calculated using COMSTAT 2.1 on the basis of the three-dimensional confocal laser scanning microscopy (CLSM) images (mean ± SEM, *n* = 5). Significance of difference with the ancestral: ns, not significant; *, *P* < 0.05; ***, *P* < 0.001 (two-tailed Student’s *t* test).

We used confocal laser scanning microscopy (CLSM) to confirm our biofilm assay and visualized the eradication of WT and DAP^R^ strain biofilms with elasnin. From the confocal images, we observed that there was a clear reduction in the cell density after treatment with elasnin for both the WT (Fig. 2a and b) and DAP^R^ MRSA (Fig. 2c and d). More importantly, the decrease in cell density was more drastic in the DAP^R^ strain compared to the WT, confirming our biofilm assay. Quantitative analysis showed that the biomass (biofilm cells) of the DAP^R^ strain was significantly reduced after the treatment of elasnin with low concentration (53% decrease in cell density), while the WT strain was only decreased slightly (15% decrease in cell density) (Fig. 2e). The maximum thickness of the biofilm was also decreased in both WT and DAP^R^ MRSA upon elasnin treatment (Fig. 2f).

### Proteomics analysis of WT and DAP^R^ MRSA biofilm cells.

**FIG 3:**
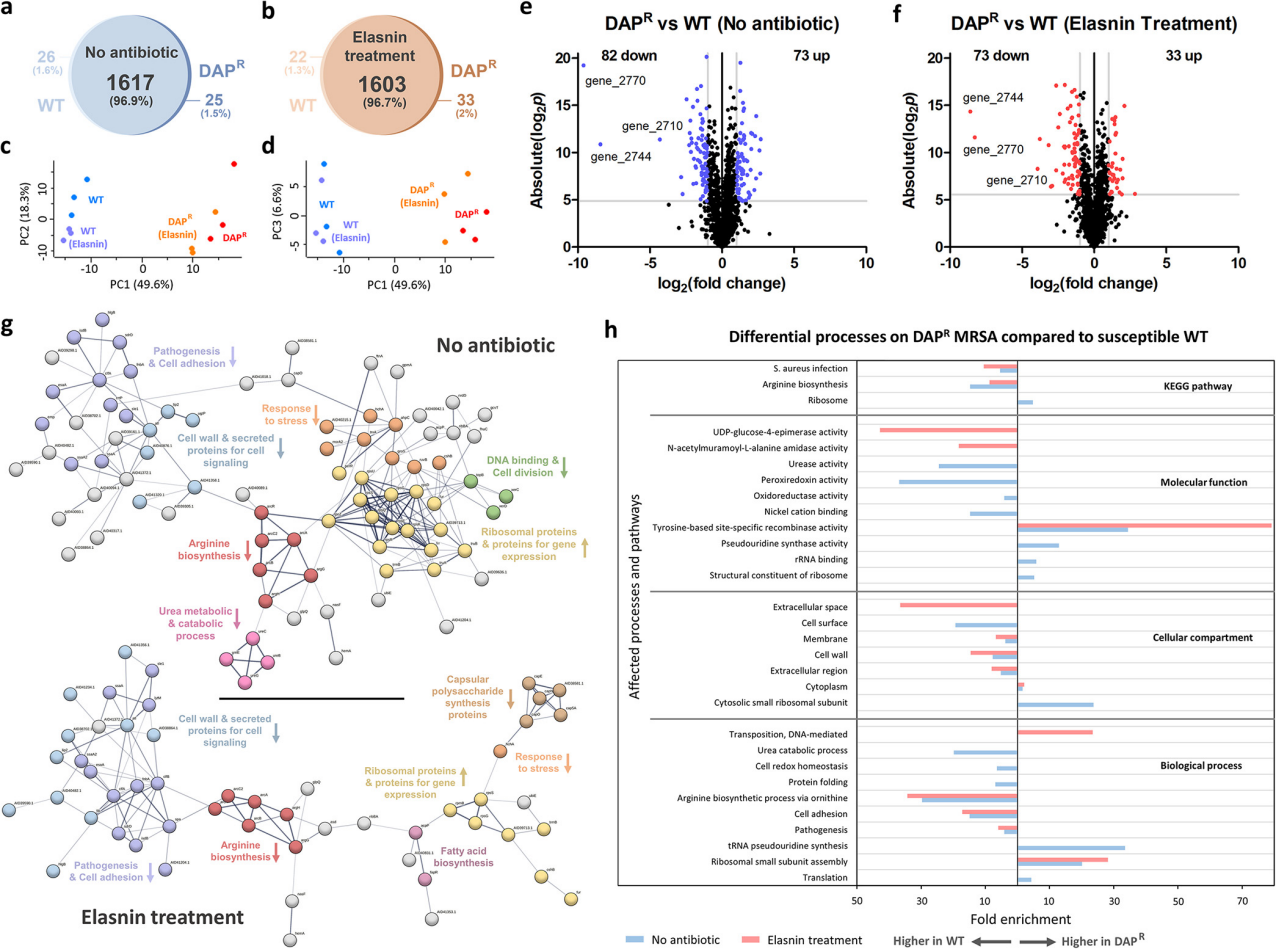
Proteome profile comparison between the DAP^R^ and the WT biofilms in the absence and presence of elasnin. (a–b) Venn diagrams for proteome comparison of DAP^R^ and the WT biofilms in the absence of antibiotic (a) and after elasnin treatment (b). (c–d) Principal-component analysis (PCA) of proteomes of the untreated WT, untreated DAP^R^, elasnin-treated WT, and elasnin-treated DAP^R^. PC1 versus PC2 (c), PC1 versus PC3 (d). (e–f) Volcano plots for DAP^R^ and the WT biofilms in the absence of antibiotic (e) and after elasnin treatment (f). Differentially expressed proteins (DEPs) are defined to be those with permutation-based false discovery rate (FDR) below 0.05, and fold change lower than 0.5 or greater than 2 (colored dots). Up-regulated proteins are those with higher expression levels on the DAP^R^ strain, while downregulated proteins are those with higher expression levels on the WT strain. (g) Protein–protein interaction network of the DEPs between the DAP^R^ and the WT biofilms, as predicted by STRING v11.0. The lines represent protein interaction (thicker lines mean higher confidence), and the dots in different colors represent different protein functions. The arrow beside the protein functions indicates whether the expression is higher (arrow pointing up) or lower (arrow pointing down) in the DAP^R^ strain compared to the WT biofilm cells. Uncharacterized proteins are not annotated and nodes without function enrichment are colored gray. (h) Gene ontology (GO) analysis and pathway enrichment study (KEGG) by DAVID of the DEPs of DAP^R^ MRSA biofilms compared to the WT in the absence of antibiotic (blue bars) and after elasnin treatment (red bars).

The superior biofilm eradication activity of elasnin toward DAP^R^ MRSA suggested that there were biofilm-specific processes that were differentially regulated in the DAP^R^ strain (compared to the WT), which made them easier to be eradicated with elasnin. To elucidate these processes, we performed shotgun proteomics on the DAP^R^ and WT biofilms (in the presence and absence of elasnin). Combining all replicates, 1,625 and 1,636 distinct proteins were identified for elasnin-treated WT and DAP^R^ biofilms respectively, whereas 1,643 and 1,642 distinct proteins were identified for untreated WT and DAP^R^ biofilms respectively (Fig. 3a and b). From the principal-component analysis (PCA) plots, we observed that while the untreated DAP^R^ and the WT biofilms were well separated from each other, the elasnin-treated cells were positioned similarly to their untreated counterparts (Fig. 3c and d). Indeed, very few proteins were found differentially expressed when comparing the cells before and after elasnin treatment (Fig. S1 in the supplemental material). The WT strain upregulated four proteins and the DAP^R^ strain upregulated three proteins in response to elasnin treatment, with one of them being shared in both strains. The WT strain upregulated ABC transporter domain-containing protein (Q2G196) by 9.2-fold, putative hemin import ATP-binding protein HrtA (Q2FVR1) by 2.5-fold, and a hypothetical protein (gene_2764) by 3.8-fold, whereas the DAP^R^ strain upregulated urease subunit beta UreB (Q2G2K6) by 5.1-fold and urease accessory protein UreF (Q2G2K7) by 2.4-fold, which were both involved in the urea degradation pathway. The protein that was upregulated in both strains was a putative multidrug ABC transporter permease YbhR (gene_2299), which was upregulated by 12- and 11-fold in the WT and DAP^R^ strains, respectively. These limited numbers of differentially regulated proteins upon elasnin treatment suggested that transport proteins played an important role in MRSA response toward elasnin treatment, while the urease degradation pathway was only upregulated in the DAP^R^ strain. As has been previously reported, the effect of elasnin treatment on the cells was most evident at ∼6 h after treatment, whereas after a prolonged treatment (>12 h), the proteome profile of the biofilm cells reverted to one that resembles the untreated cells. This is likely because at an extended time, most of the treated biofilm cells that exhibited physiological changes were already released to the medium. Those analyzed by proteomics were the cells left behind, which should be located deeper in the biofilm with possibly less elasnin exposure (7). In our case, the biofilm cells were treated with a long treatment period (18 h), which may explain the similar profiles of the treated and untreated cells. Therefore, in this study, we focused on comparing the proteome of the DAP^R^ and WT biofilm cells, which will tell us why the DAP^R^ biofilms were more readily eradicated with elasnin, rather than comparing the proteome of the biofilm cells before and after elasnin treatment, which was already reported elsewhere (7).

Fig. 3e and f show the volcano plots of fold changes against *P* values (two-tailed Student's *t* test) along with the number of differentially expressed proteins (DEPs), highlighting the proteins in the DAP^R^ biofilm with different expression levels compared to the WT in the absence and presence of elasnin, respectively. A total of 155 proteins (73 proteins upregulated and 82 proteins downregulated) were differentially expressed in the DAP^R^ strain compared to the WT in the absence of antibiotic, whereas 106 proteins (33 proteins upregulated and 73 proteins downregulated) were differentially expressed in the DAP^R^ strain compared to the WT in the presence of elasnin. The list of DEPs is available in Table S1, and the protein-protein interaction networks of the DEPs are depicted in Fig. 3g. In the absence of elasnin, the expression of ribosomal proteins was higher in the DAP^R^ biofilm, while the expression of proteins for arginine biosynthesis, urea cycle, response to stress, cell signaling, pathogenesis, and cell adhesion was lower compared to the WT. After elasnin treatment, we observed a smaller number of processes whose expression levels differed between the two strains. While we still spotted the lower expression of proteins for arginine biosynthesis, cell signaling, pathogenesis, and cell adhesion, the lower expression of proteins for the urea cycle and those that act as a response to stress in the DAP^R^ biofilm cells were no longer observed, indicating that they upregulated these proteins after elasnin treatment to a similar level as those in the WT cells. This was consistent with the observed upregulation of proteins for urea degradation (UreB and UreF) on the DAP^R^ strain upon elasnin treatment (Fig. S1), whereas the WT strain did not differentially express these proteins.

### DAP^R^ MRSA has a lower expression of proteins for pathogenesis and cell adhesion compared to the WT, which are important for biofilm development.

Similarly, from the Gene Ontology (GO) and pathway analysis, in the absence of elasnin we observed that the DAP^R^ strain has a higher expression of ribosomal proteins, pseudouridine synthase activity, and proteins for rRNA binding compared to the WT, which were all related to gene expression (Fig. 3h). Both in the absence and presence of elasnin, the DAP^R^ strain has a lower expression of membrane and cell wall proteins, and proteins for arginine biosynthesis, pathogenesis, and cell adhesion, indicating that these processes were already differentially regulated in the DAP^R^ strain even before exposure to elasnin, and elasnin treatment also did not alter the regulation of these processes. Interestingly, although the tyrosine-based-site-specific recombinase activity was expressed higher in the DAP^R^ strain even in the absence of elasnin, its fold enrichment in GO analysis drastically increased upon treatment with elasnin, indicating that there are much more proteins with this molecular function that were expressed higher upon elasnin treatment. On the other hand, there are some processes that were altered in the DAP^R^ strain only upon exposure to elasnin, such as the higher expression of proteins related to transposition, UDP-glucose-4-epimerase activity, N-acetylmuramoyl-L-alanine amidase activity, and proteins in the extracellular space. These differential regulations of processes upon elasnin treatment in the two strains might be related to the observed difference in their biofilm eradication.

**FIG 4:**
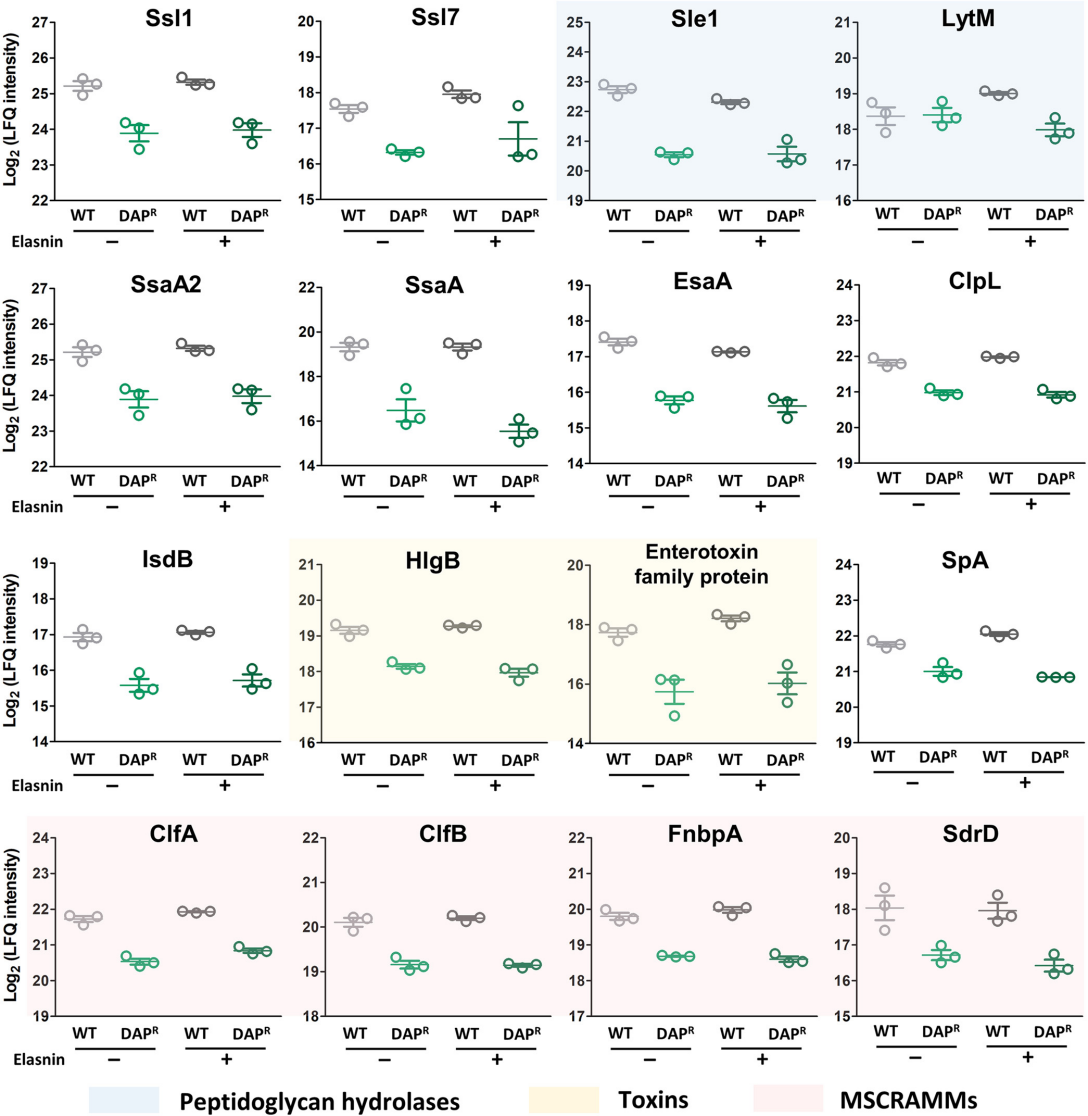
Expression level of proteins involved in pathogenesis and cell adhesion across the DEPs of the WT and DAP^R^ strain. Those that are highlighted with blue colors are peptidoglycan hydrolases, yellow colors are toxins, and red colors are microbial surface components recognizing adhesive matrix molecules (MSCRAMMs), which mediate the initial attachment of S. aureus to host tissue, hence providing a critical step for infection establishment.

A large array of virulence factors and secreted molecules participate in the typical process of S. aureus biofilm formation and development (18–20). These include adhesive surface proteins (e.g., fibronectin-binding proteins FnbpA and FnbpB, clumping factors ClfA and ClfB), degradative enzymes (e.g., SspA, SspB), autolysins (e.g., LytM and Atl), extracellular polymeric substances (wall teichoic acids, polysaccharide intracellular adhesin, extracellular DNA), toxins (e.g., Hla and HlgAB), and many others (21–23). Indeed, we observed that the expression of these proteins was lower in the DAP^R^ strain. The expression profile of the DEPs involved in pathogenesis and cell adhesion across both the WT and DAP^R^ strains is shown in Fig. 4, where we spotted that microbial surface components recognizing adhesive matrix molecules (MSCRAMMs) such as clumping factors A and B (ClfA, ClfB), fibrinogen binding protein A (FnbpA), and serine-aspartate repeat-containing protein SdrD, were expressed lower in the DAP^R^ biofilm cells compared to the WT. Other proteins that were expressed lower in the DAP^R^ biofilm cells include autolysins such as N-acetylmuramoyl-L-alanine amidase Sle1, glycyl-glycine endopeptidase LytM, and Atl, which are peptidoglycan hydrolases involved in cell wall formation during cell division, toxins such as gamma-hemolysin component B (HlgB) and enterotoxin family protein, and virulence factors such as staphylococcal superantigen-like 1 and 7 (Ssl1 and Ssl7), which are virulence mediators that proteolytically cleave host proteins, type VII secretion system accessory factor EsaA, which acts a secretion platform across cytoplasmic membrane in the host, iron-regulated surface determinant protein B (IsdB) which is a cell wall-anchored surface receptor, and staphylococcal protein A (SpA), which plays a key role in the inhibition of the host innate and adaptive immune responses. Besides, we also observed that extracellular matrix binding protein Emp, one of the secretable expanded repertoire adhesive molecules (SERAMs), was expressed lower by 3.5-fold in the DAP^R^ strain compared to the WT. Since all of these proteins are important for biofilm development, this might explain why the DAP^R^ biofilm cells were easier to be eradicated with elasnin. Besides, the observation that the DAP^R^ strain has lower expression of key proteins for biofilm development was also consistent with the previously reported lower biofilm formation of the DAP^R^ strain compared to the WT MRSA (16).

A previous study that investigated the transcriptome and proteome changes of MRSA biofilm cells upon elasnin treatment showed that in addition to the membrane and extracellular proteins, elasnin treatment led to the downregulation of genes and proteins involved in cell division, staphylococcus infection, and pathogenesis (7). These genes/proteins include those for the EPS production (IcaA, IcaB, and IcaC), murein hydrolases and autolysins (Atl, LytM, LytR, and CidA), serine protease (SspA, SspB, SspC, SspP, and SplB), toxins (Hld, HlgB, HlgC, and Hly), secreted virulence factors (EsxA), and adhesins (FnbpA, ClfB, SdrD, and Emp), which we also observed to be expressed lower in the DAP^R^ strain compared to the WT biofilm cells. These suggested that the expression of these proteins was important for the cells to withstand biofilm eradication by elasnin, but instead, they were expressed lower in the DAP^R^ strain, consistent with their higher elasnin-induced biofilm eradication.

Our findings pointed out the clinical potentials of elasnin. Aside from the effectiveness of elasnin in eradicating DAP^R^ MRSA biofilms through the larger downregulation of important proteins for biofilm development, it can also kill DAP^R^ planktonic cells to a similar extent to the WT strain under prolonged lethal-dose treatment. DAP^R^ MRSA strains have become prevalent in clinics and often formed biofilms in clinical devices, which could not be eradicated by antibiotics such as DAP and VAN (16). Therefore, the discovery of novel antibiofilm agents that can eradicate these notorious resistant MRSA strains would be of great interest. Overall, this study highlights the potential use of elasnin in clinical settings through its superior biofilm eradication activity against DAP^R^ MRSA biofilms compared to the WT and elucidated the processes governing this superiority, which would be useful for follow-up studies focusing on *in vivo* applications of elasnin as an antibiotic or drug adjuvant.

## MATERIALS AND METHODS

### Bacterial strains.

In this study, methicillin-resistant S. aureus (MRSA) ATCC 43300 was used as the wild-type strain. The daptomycin-resistant strain (DAP^R^) with single point mutation on the *mprF* gene (T345A) was obtained through laboratory evolution experiments from our previous study (16).

### Elasnin preparation and treatment in planktonic cells.

The elasnin was prepared according to a previously described protocol (5, 8). Briefly, Streptomyces mobaraensis DSM 40847 was incubated for 5 days, and the culture broth was extracted with ethyl acetate. Pure elasnin was isolated by reversed-phase high-performance liquid chromatography (HPLC) and dissolved in dimethyl sulfoxide (DMSO).

To generate the time-kill curves upon elasnin treatment, overnight cultures of MRSA were prepared by incubating cells from frozen stock in cation-adjusted Mueller-Hinton (MH) broth (supplemented with 50 mg/L Ca^2+^) at 37°C at 250 rpm. The next day, cells were regrown by incubating a 1:1,000 diluted overnight culture in fresh MH broth at 37°C until it reached exponential phase (OD_600_ of ∼0.1). The exponential phase cultures were treated with elasnin (50 μg/mL) for 21 h, and the number of survivors was counted at specific time points by serially diluting cultures in MH broth, plating 100 μL on MH agar and spread plates.

### Disc diffusion assay.

The MICs of MRSA cells toward elasnin were measured by broth macrodilution and disc diffusion assay. For broth macrodilution, the MIC was determined by incubating ∼5⋅10^5^ exponential phase bacteria in MH broth overnight with different antibiotics concentrations. The MIC value was determined as the lowest concentration without growth. The disc diffusion test was performed according to the standard EUCAST susceptibility test guideline where inoculum suspension equivalent to a 0.5 McFarland standard (1–2⋅ 10^8^ CFU/mL) was spread on MH agar applied with an antimicrobial disk containing 40 μg elasnin, and incubated at 35°C for 20 h (24).

### Biofilm assay.

Biofilm assay was performed as previously described (25, 26). To see the biofilm inhibition, an overnight culture of WT and DAP^R^ MRSA was diluted into approximately 10^7^ CFU/mL with Lysogeny broth (LB) and 0.5% glucose and treated with various concentrations of elasnin in 96-well cell culture plates. Plates were then incubated at 37°C for 24 h and rinsed twice with 1 × PBS (phosphate-buffered saline) to remove nonadhering and planktonic cells. After rinsing, MTT staining assay was conducted to measure viable cells in the biofilms since MTT can react with activated succinate dehydrogenase in viable cell mitochondria to form blue-violet formazan, which can be measured at 570 nm after dissolving in DMSO. Biofilm inhibition (%) is calculated by comparing the OD_570_ values of the antibiotic-treated cells with the OD_570_ values of the cells grown without antibiotics. To see the biofilm eradication, an overnight culture of WT and DAP^R^ MRSA was incubated for 24 h in 96-well cell culture plates to form biofilms (without the addition of elasnin). The formed biofilm was rinsed twice with 1 × PBS and challenged with elasnin at a series of concentrations and incubated for another 24 h at 37°C. After incubation, each well was rinsed twice with 1 × PBS, and the MTT assay was conducted to measure viable cells in the remaining biofilm. Biofilm eradication (%) is calculated by comparing the OD_570_ values of the antibiotic-treated biofilms with the OD_570_ values of the biofilms without antibiotic treatment. The lowest concentrations of elasnin that resulted in decreases of at least 90% and 50% in OD_570_ of the WT and DAP^R^ MRSA were recorded as minimum biofilm inhibitory concentration (MBIC) and minimum biofilm eradication concentration (MBEC), respectively.

### Confocal laser scanning microscopy (CLSM) observation of biofilm cells under elasnin treatment.

Biofilms were grown on glass cover slides as those described for the MBEC assay with 0.625 μg/mL elasnin. Treated biofilms were then rinsed twice with 1 × PBS and stained with FilmTracer FM 1-43 green biofilm cell stain at room temperature for 30 min in the dark. A Zeiss LSM710 Confocal Microscope was employed to observe cells in the biofilm at 488 nm.

### Sample preparation for proteomics.

The WT and DAP^R^ MRSA were incubated for 24 h in 96-well cell culture plates to form biofilms. The formed biofilm was rinsed twice with 1 × PBS and treated with elasnin (0.625 μg/mL) for 18 h. The long treatment time was chosen to ensure that we profiled the biofilm cells with similar progression toward elasnin treatment, rather than those that were still in transition from biofilm to planktonic. Biofilm cells that were not treated with elasnin were collected as a control. For all samples, three biological replicates were performed including the control samples.

The subsequent processing of the samples follows the same protocol from our previous study (8). Briefly, the cell pellet was suspended in 350 μL of lysis buffer (8M Urea, 50 mM Tris-HCl pH 8.0), frozen in liquid nitrogen, and sonicated for 15 min. The sample was centrifuged, and an aliquot of the sample was taken for bicinchoninic acid protein assay. After protein quantification, the sample was reduced by dithiothreitol (DTT) to a 0.1 M final concentration at 37°C for 1 h. Next, 100 μg of proteins were transferred to an Amicon filter device and centrifuged, and the retained proteins were alkylated with iodoacetamide (IAA, 50 mM in exchange buffer) in the dark for 20 min, and then centrifuged again. To dilute the urea concentration, 250 μL of 50 mM ammonium bicarbonate was added to the filter device and centrifuged, and this step was repeated once. Proteins were digested by sequencing-grade modified trypsin (1:50 wt/wt) for 12 h at 37°C, and then the sample was acidified with 10% formic acid to a final concentration of 0.1% (vol/vol), followed by 5 min centrifugation. The samples were desalted by C18 reverse-phase ZipTip and dried with SpeedVac for 20 min.

### Liquid chromatography (LC) – TimsTOF Pro mass spectrometer (MS).

Similarly, the parameters and settings for the LC-MS follow the same protocol from our previous study (8). Briefly, the samples were reconstituted in 25 μL water:acetonitrile:formic acid in a 97.9:2:0.1 ratio (vol/vol/vol), and a volume of 1 μL (200 ng of the protein digest) was processed through Bruker nanoElute Ultra-High-Performance Liquid Chromatography (UHPLC) coupled to a hybrid trapped ion mobility-quadrupole time-of-flight mass spectrometer (TimsTOF Pro) via a nano-electrospray ion source. The mobile phase composition is 0.1% formic acid in water for solvent A, and 0.1% formic acid in acetonitrile for solvent B. The gradient was applied as follows: from 2% to 5% of solvent B for the first 0.5 min, from 5% to 30% of solvent B for 26.5 min, from 30% to 95% of solvent B for 0.5 min, keeping 95% of solvent B for 0.5 min, and finally decreasing from 95% to 2% of solvent B for 0.1 min. Two min equilibration with 2% of solvent B was applied before the next injection.

For the TimsTOF Pro mass spectrometer (MS), we set the accumulation and ramp time to 100 ms each and recorded mass spectra in the range from *m/z* 100 to 1700 using the positive electrospray mode. The ion mobility was scanned from 0.85 to 1.30 Vs/cm^2^, the quadrupole isolation width was set to 2 Th for *m/z* < 700 and 3 Th for *m/z* > 700, and the collision energy was linearly increased from 27 eV to 45 eV as a function of increasing ion mobility. Low-abundance precursor ions with an intensity above a threshold of 2,500 counts but below a target value of 20,000 counts were repeatedly scheduled and otherwise dynamically excluded for 0.4 min.

### Database searching and label-free quantification of proteomics data.

The database searching and quantification of proteomics data follow the same protocol from our previous study (8). Briefly, the raw data were processed by MaxQuant v1.6.17.0 (27, 28) with default parameters except the following. Discard unmodified counterpart peptides was set to false, write msScans table was set to true, and match between runs was set to true. For label-free quantification (LFQ), the minimum ratio count was set to 2, and separate LFQ in parameter groups was set to true. For the database searching, a custom database was generated by converting the genome sequence of MRSA ATCC 43300 into a protein database using GeneMark (29) (version 3.25) gene prediction tool. The proteins were subsequently annotated using BLASTp (version 2.7.1) from NCBI using MRSA NCTC 8325 as the protein database. The sequences of common contaminants such as trypsin and human keratins were then added to the database. Perseus v1.6.15.0 (30) was used to process the subsequent data from MaxQuant. Briefly, protein group LFQ intensities were log_2_-transformed, missing values were imputed from the normal distribution, and log ratios were calculated as the difference in the average log_2_ LFQ intensity values between experimental and control groups. Two-tailed Student's *t* test calculations were used in statistical tests. A protein is considered as differentially expressed if its fold change is higher or lower than ±2-fold and permutation-based false discovery rate (FDR) ≤ 0.05.

Perseus was used to visualize the proteomic data using principal-component analysis of the log_2_ LFQ intensity values. To highlight potentially important proteins among the differentially expressed proteins, STRING version 11.0 (31) was used to predict the protein–protein interactions and to visualize the interactions. Finally, DAVID (Database for Annotation, Visualization and Integrated Discovery) version 6.8 (32) was used for gene ontology (GO) and Kyoto Encyclopedia of Genes and Genomes (KEGG) pathway analysis.

### Data availability.

The mass spectrometry proteomics data have been deposited to ProteomeXchange via the PRIDE repository with the data set identifier PXD024005.

## References

[1] Omura S, Ohno H, Saheki T, Yoshida M, Nakagawa A. 1978. Elasnin, a new human granulocyte elastase inhibitor produced by a strain of Streptomyces. Biochem Biophys Res Commun 83:704–709. doi:10.1016/0006-291x(78)91046-x. 697852

[2] Ohno H, Saheki T, Awaya J, Nakagawa A, Omura S. 1978. Isolation and characterization of elasnin, a new human granulocyte elastase inhibitor produced by a strain of Streptomyces. J Antibiot (Tokyo) 31:1116–1123. doi:10.7164/antibiotics.31.1116. 721707

[3] Groutas W, Abrams W, Carroll R, Moi M, Miller K, Margolis M. 1984. Specific inhibition of human leukocyte elastase by substituted alpha-pyrones. Experientia 40:361–362. doi:10.1007/BF01952552. 6562017

[4] Groutas WC, Stanga MA, Brubaker MJ, Huang TL, Moi MK, Carroll RT. 1985. Substituted 2-pyrones, 2-pyridones, and other congeners of elasnin as potential agents for the treatment of chronic obstructive lung diseases. J Med Chem 28:1106–1109. doi:10.1021/jm00146a023. 3848491

[5] Long L, Wang R, Chiang HY, Ding W, Li Y-X, Chen F, Qian P-Y. 2021. Discovery of antibiofilm activity of elasnin against marine biofilms and its application in the marine antifouling coatings. Mar Drugs 19:19. doi:10.3390/md19010019. 33466541 PMC7824865

[6] Mishra R, Panda AK, De Mandal S, Shakeel M, Bisht SS, Khan J. 2020. Natural anti-biofilm agents: strategies to control biofilm-forming pathogens. Front Microbiol 11:566325. doi:10.3389/fmicb.2020.566325. 33193155 PMC7658412

[7] Long L, Sulaiman JE, Xiao Y, Cheng A, Wang R, Malit JJ, Wong WC, Liu W, Li Y-X, Chen F, Lam H, Qian P-Y. 2021. Mode of action of elasnin as biofilm-formation eradicator of methicillin-resistant *Staphylococcus aureus*. ResearchSquare. doi:10.21203/rs.3.rs-752510/v1. PMC9393526 36003935

[8] Sulaiman JE, Long L, Qian P-Y, Lam H. 2022. Proteomics and transcriptomics uncover key processes for elasnin tolerance in methicillin-resistant *Staphylococcus aureus*. mSystems 7:e01393-21. doi:10.1128/msystems.01393-21. PMC8788329 35076266

[9] Liu C, Bayer A, Cosgrove SE, Daum RS, Fridkin SK, Gorwitz RJ, Kaplan SL, Karchmer AW, Levine DP, Murray BE, J Rybak M, Talan DA, Chambers HF, Infectious Diseases Society of America. 2011. Clinical practice guidelines by the Infectious Diseases Society of America for the treatment of methicillin-resistant *Staphylococcus aureus* infections in adults and children. Clin Infect Dis 52:e18–e55. doi:10.1093/cid/ciq146. 21208910

[10] Sass P, Berscheid A, Jansen A, Oedenkoven M, Szekat C, Strittmatter A, Gottschalk G, Bierbaum G. 2012. Genome sequence of *Staphylococcus aureus* VC40, a vancomycin-and daptomycin-resistant strain, to study the genetics of development of resistance to currently applied last-resort antibiotics. J Bacteriol 194:2107–2108. doi:10.1128/JB.06631-11. 22461548 PMC3318483

[11] Wang G, Hindler JF, Ward KW, Bruckner DA. 2006. Increased vancomycin MICs for *Staphylococcus aureus* clinical isolates from a university hospital during a 5-year period. J Clin Microbiol 44:3883–3886. doi:10.1128/JCM.01388-06. 16957043 PMC1698298

[12] Appelbaum P. 2006. The emergence of vancomycin‐intermediate and vancomycin‐resistant *Staphylococcus aureus*. Clin Microbiol Infect 12:16–23. doi:10.1111/j.1469-0691.2006.01344.x. 16445720

[13] Mangili A, Bica I, Snydman D, Hamer D. 2005. Daptomycin-resistant, methicillin-resistant *Staphylococcus aureus* bacteremia. Clin Infect Dis 40:1058–1060. doi:10.1086/428616. 15825002

[14] Van Hal S, Paterson DL, Gosbell IB. 2011. Emergence of daptomycin resistance following vancomycin-unresponsive *Staphylococcus aureus* bacteraemia in a daptomycin-naive patient—a review of the literature. Eur J Clin Microbiol Infect Dis 30:603–610. doi:10.1007/s10096-010-1128-3. 21191627

[15] Capone A, Cafiso V, Campanile F, Parisi G, Mariani B, Petrosillo N, Stefani S. 2016. In vivo development of daptomycin resistance in vancomycin-susceptible methicillin-resistant *Staphylococcus aureus* severe infections previously treated with glycopeptides. Eur J Clin Microbiol Infect Dis 35:625–631. doi:10.1007/s10096-016-2581-4. 26815434

[16] Sulaiman JE, Long L, Wu L, Qian P-Y, Lam H. 2021. Comparative proteomic investigation of multiple methicillin-resistant *Staphylococcus aureus* strains generated through adaptive laboratory evolution. iScience 24:102950. doi:10.1016/j.isci.2021.102950. 34458699 PMC8377494

[17] Donlan RM. 2001. Biofilms and device-associated infections. Emerg Infect Dis 7:277–281. doi:10.3201/eid0702.010226. 11294723 PMC2631701

[18] Otto M. 2018. Staphylococcal biofilms. Microbiol Spectr 6:27. doi:10.1128/microbiolspec.GPP3-0023-2018. PMC6282163 30117414

[19] Antunes LCM, Ferreira RB. 2011. Biofilms and bacterial virulence. Rev Med Microbiol 22:12–16. doi:10.1097/MRM.0b013e3283410d22.

[20] Alves PM, Al-Badi E, Withycombe C, Jones PM, Purdy KJ, Maddocks SE. 2018. Interaction between *Staphylococcus aureus* and *Pseudomonas aeruginosa* is beneficial for colonisation and pathogenicity in a mixed biofilm. Pathog Dis 76:fty003. doi:10.1093/femspd/fty003. 29342260

[21] Bose JL, Lehman MK, Fey PD, Bayles KW. 2012. Contribution of the *Staphylococcus aureus* Atl AM and GL murein hydrolase activities in cell division, autolysis, and biofilm formation. 7:e42244. doi:10.1371/journal.pone.0042244. PMC3409170 22860095

[22] Heilmann C, Hussain M, Peters G, Götz F. 1997. Evidence for autolysin‐mediated primary attachment of *Staphylococcus epidermidis* to a polystyrene surface. Mol Microbiol 24:1013–1024. doi:10.1046/j.1365-2958.1997.4101774.x. 9220008

[23] Schlag M, Biswas R, Krismer B, Kohler T, Zoll S, Yu W, Schwarz H, Peschel A, Götz F. 2010. Role of staphylococcal wall teichoic acid in targeting the major autolysin Atl. Mol Microbiol 75:864–873. doi:10.1111/j.1365-2958.2009.07007.x. 20105277

[24] Jorgensen JH, Turnidge JD. 2015. Susceptibility test methods: dilution and disk diffusion methods, p 1253–1273, *In* Jorgensen JH, Carroll KC, Funke G, Pfaller MA, Landry ML, Richter SS, Warnock DW (ed), Manual of clinical microbiology, 11th ed. Wiley, New York, NY.

[25] Yin Q, Liang J, Zhang W, Zhang L, Hu Z-L, Zhang Y, Xu Y. 2019. Butenolide, a marine-derived broad-spectrum antibiofilm agent against both Gram-positive and Gram-negative pathogenic bacteria. Mar Biotechnol (NY) 21:88–98. doi:10.1007/s10126-018-9861-1. 30612218 PMC6394721

[26] Nair S, Desai S, Poonacha N, Vipra A, Sharma U. 2016. Antibiofilm activity and synergistic inhibition of *Staphylococcus aureus* biofilms by bactericidal protein P128 in combination with antibiotics. Antimicrob Agents Chemother 60:7280–7289. doi:10.1128/AAC.01118-16. 27671070 PMC5119008

[27] Cox J, Mann M. 2008. MaxQuant enables high peptide identification rates, individualized ppb-range mass accuracies and proteome-wide protein quantification. Nat Biotechnol 26:1367–1372. doi:10.1038/nbt.1511. 19029910

[28] Tyanova S, Temu T, Cox J. 2016. The MaxQuant computational platform for mass spectrometry-based shotgun proteomics. Nat Protoc 11:2301–2319. doi:10.1038/nprot.2016.136. 27809316

[29] Lukashin AV, Borodovsky M. 1998. GeneMark.hmm: new solutions for gene finding. Nucleic Acids Res 26:1107–1115. doi:10.1093/nar/26.4.1107. 9461475 PMC147337

[30] Tyanova S, Temu T, Sinitcyn P, Carlson A, Hein MY, Geiger T, Mann M, Cox J. 2016. The Perseus computational platform for comprehensive analysis of (prote)omics data. Nat Methods 13:731–740. doi:10.1038/nmeth.3901. 27348712

[31] Szklarczyk D, Morris JH, Cook H, Kuhn M, Wyder S, Simonovic M, Santos A, Doncheva NT, Roth A, Bork P. 2017. The STRING database in 2017: quality-controlled protein–protein association networks, made broadly accessible. Nucleic Acids Res 45:D362–D368. doi:10.1093/nar/gkw937. 27924014 PMC5210637

[32] Huang DW, Sherman BT, Lempicki RA. 2009. Systematic and integrative analysis of large gene lists using DAVID bioinformatics resources. Nat Protoc 4:44–57. doi:10.1038/nprot.2008.211. 19131956

