# Supplementary material for: Elasnin Effectively Eradicates Daptomycin-Resistant Methicillin-Resistant Staphylococcus aureus Biofilms
Source: Microbiol Spectr. 2022 Feb 23;10(1):e02320-21. doi: 10.1128/spectrum.02320-21 (PMC8865424; doi:10.1128/spectrum.02320-21)
Supplement: SUPPLEMENTAL FILE 1 — Supplemental material. Download SPECTRUM02320-21_Supp_1_seq9.pdf, PDF file, 0.1 MB [file spectrum02320-21_supp_1_seq9.pdf]

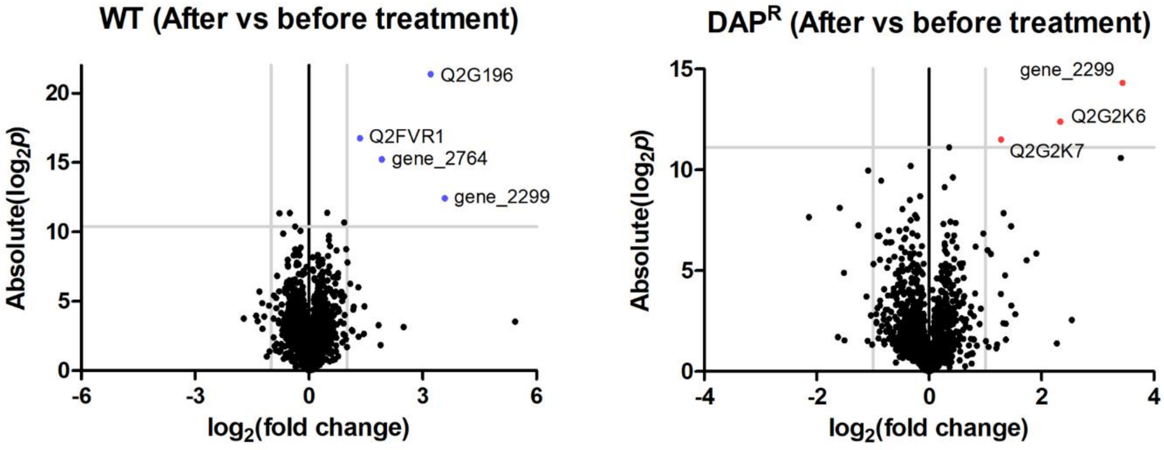

Fig. S1. Volcano plots for WT and DAP^{R} biofilms after 18 h of elasnin treatment compared to those before treatment. Differentially expressed proteins (DEPs) are defined to be those with false discovery rate (FDR) below 0.05, and absolute fold change greater than 2, corresponding to the colored dots. The protein IDs of the DEPs are shown.
